# Exploring the Relationship (and Power Dynamic) Between Researchers and Public Partners Working Together in Applied Health Research Teams

**DOI:** 10.3389/fsoc.2019.00020

**Published:** 2019-03-29

**Authors:** Gill Green, Tracey Johns

**Affiliations:** School of Health and Social Care, University of Essex, Colchester, United Kingdom

**Keywords:** public involvement, co-production of research, PPI in research, partnerships with the public, applied health research

## Abstract

Public involvement in applied health research in the UK has become a pre-requisite for receiving funding from some bodies including the National Institute of Health Research. However, much of this involvement has been criticized as being tokenistic with an unequal power dynamic whereby the public voice is consulted but may be ignored. To redress this imbalance more participatory methods of involvement, such as co-production have emerged. This paper explores the relationship and power dynamic between researchers and public partners through the thematic analysis of interviews with fourteen researchers and six public contributors who were involved in projects that were identified as having many features associated with inclusive co-produced research. Public involvement was valued but the integration of scientific and lay knowledge on an equal basis was problematic. In practice, “co-opted relationships” were most common whereby public partners were slotted into a designated role created for them by the researcher/research team. There were though some examples of more equal partnerships being established to share power and decision-making including two cases where the research idea was initiated by the public partner. However, establishing an equal relationship and sharing power was constrained by the hierarchical nature of applied health research as well as issues around governance and accountability. Specifically, the positivist paradigm that predominates in applied health research and tends to privilege classically scientific ways of thinking, was a barrier to experiential knowledge being equally valued. This demonstrates the challenges inherent in establishing equal relationships and suggests that a transformation of research practices, culture and hierarchies is required for power sharing to become a reality. Specifically, the culture of applied health research needs to embrace more democratic participatory approaches, such as those used in research originating from the service user movement, as it is within these ways of working that public partners can more readily share power.

## Introduction

There is a proliferation of experiential or lay expertise in current knowledge societies (see Lambert and Rose, 1996; Grundmann, 2017) and a growing number of well-qualified citizens are knowledgeable and interested in issues that were previously the exclusive domain of professionals and scientists. This is pronounced in the world of health research where people with a health condition have a wealth of experiential expertise to draw upon. Public involvement (PI) in applied health research has increased dramatically in many parts of the world (Evans, 2014; Wicks et al., 2018), and in the UK, engagement with the public in constructing a research proposal is a pre-requisite for obtaining funding from the National Institute of Health Research (NIHR). By the term “public” we include members of the community, patients, carers, and people who use health and social care services. Our definition of applied health research includes research into treatments, devices, and procedures, and translation into practice to improve care. This includes health services research but not laboratory research. A PI infrastructure has subsequently developed whereby research organizations and funders develop relationships with members of the public, patients and charitable health organizations to ensure that PI is embedded in research (Department of Health, 2015).

However, this movement has been critiqued for being conceptually and theoretically vague (Madden and Speed, 2017), having evolved from a range of rationales, values, epistemologies, and political movements (Paylor and McKevitt, in press). PI has its theoretical roots in two entirely separate traditions, one based on human rights whereby citizens have rights and responsibilities and the other upon consumerist neo-liberalism (Taylor, 2007). There is consensus that the consumerist managerial model has tended to dominate PI and delineate the scope of involvement in that the role of public partners is primarily consultative; they provide feedback to the researchers but do not drive the project (Beresford, 2002). As a result the democratizing and emancipatory potential of PI as conceptualized through Paolo Freire's (1921–1997) process of “conscientization” (Freire, 1993) is limited. Furthermore, participation without a redistribution of power serves to maintain rather than challenge the status quo (Arnstein, 1969).

Green (2016) reviewed the landscape of PI in applied health research to identify the extent to which there is evidence of power shifting from the scientific research community to the public. She found that whilst patients and the public are a key part of the applied health research infrastructure in the UK and their contribution is evident in a range of decision-making processes from identifying priority areas for commissioning research to making decisions about aspects of research design and which projects are funded, there has not been a transformation of the social relations or power dynamic between the scientific research community and the public.

However, the democratizing pressure that comes from service users organizations and the conscientization process itself, whereby participation fuels a thirst for further involvement, have resulted in a growing appetite among the public and some researchers for the public to have a stronger voice. Thus, more inclusive methods of involvement, such as co-production, have become firmly identified as a way to strengthen PI in applied health research (Department of Health, 2015; Staniszewska et al., 2018). To provide clarity and guidance about co-production, INVOLVE, the organization that drives PI in research for NIHR, produced a report which defines co-production as “an approach in which researchers, practitioners and the public work together, sharing power and responsibility from the start to the end of the project, including the generation of knowledge” (Hickey et al., 2018). In this scenario, public contributors are regarded as assets and active agents in order to share power and co-produce research (Department of Health, 2015). Putting this into practice can though be problematic. One example is a Patient Led Research hub that has been established by a clinical trials unit to enable patients and the public not only to propose research questions, but to design, initiate, and deliver their own research with support from research professionals. However, some researchers are skeptical of this approach and the focus of the patient led hub rarely aligns with existing funding streams (Mader et al., 2018).

“Co-production” is closely associated with and builds on traditions of participatory research as well as that originating from the service user movement (Beresford, 2019). The value attributed to such approaches in academic cultures is variable and they have not traditionally been dominant in applied health research. Notwithstanding the growth of qualitative research and sociological approaches in applied health research, including health services research, which sometimes use participatory methods, the biomedical model based on a positivist approach remains dominant. This places meta analyses followed by randomized controlled trials (RCTs) at the top of the methodological pyramid and qualitative studies and anecdotal evidence at the bottom. There are critiques of the hierarchy of evidence, as RCTs are only appropriate to evaluate the effectiveness of interventions. They cannot assess acceptability to the patient which is why process evaluations using qualitative methods are now embedded into many trials (Moore et al., 2015). Nevertheless, the positivist approach tends to remain dominant in most applied health research. This privileges scientific knowledge over lay understanding, which creates significant difficulties in accessing and articulating views of publics (Taylor, 2007). The value of lay experiential knowledge sits uneasily with the predominantly positivist approach of biomedical research which is based on controlled conditions, isolated variables and measurement requiring scientific expertise.

For experiential knowledge to have full value, there must be a space in which both expert and lay knowledge can interact with each other on an equal basis and this space has yet to materialize in applied health research (Gibson et al., 2012). However, it is not entirely clear what constitutes an “equal basis” as the integration of experiential knowledge with scientific expertise is complex.

Firstly, from a theoretical perspective both types of knowledge are often synergistic and should not necessarily be seen as competing (Callon, 1999). There is no fixed boundary between lay and expert knowledge, rather if has been conceptualized as a continuum of different forms of knowledge (McClean and Shaw, 2005) and researchers with lived experience combine both types of knowledge. It is the differing perspectives that both “experts” and “publics” bring that it seen as so valuable in producing research that is both high quality and relevant to the real lives of patients and the public. However, in practice, disparities between research knowledge produced through the research process and knowledge based on lived experience can create binary polarities.

Secondly, operating on an “equal basis” does not equate to researchers and public partners having equal power in all decision-making. They are part of a research team which is likely to include different grades of research staff (with one being the designated “Chief investigator”), clinicians and managers as well as public members. Decision-making will vary according to the expertise required, e.g., determining the size of the sample needs the expertise of a statistician rather than someone who has little understanding of a power calculation. What seems key is sharing power and for each member of the research team to feel empowered to make decisions in areas where they have the requisite expertise (Hickey et al., 2018). Thus, to operate on an “equal basis” a public partner would expect to be the dominant voice in decisions about aspects of the research related to the patient experience but not in decisions related to the scientific method.

Thirdly, failure to establish an equal relationship is often attributed to the researcher not fully recognizing the value of lay knowledge and being unwilling to share power (see Brett et al., 2014; Wicks et al., 2018). Whilst this may sometimes be evident, a focus on the individual researcher detracts from the broader contextual constraints within which PI operates. The consumerist managerialism which dominates the implementation of PI in applied health research may place limits on the opportunities for collaborative democratic approaches and also explain the negative attitudes of some researchers (Paylor and McKevitt, in press).

In an attempt to define what constitutes more inclusive methods of involvement, key principles of co-production have been identified as: sharing of power; including all perspectives and skills; respecting and valuing the knowledge of all those working together on the research; reciprocity; building and maintaining relationships (Hickey et al., 2018). Whilst these seem relatively straightforward, the challenge is the translation into practice. How these principles can be achieved amidst the maelstrom of competing priorities to complete a research project is not well-understood.

Furthermore, much of the evidence about the integration of expert and experiential knowledge in applied health research is based on critical/ theoretical review of policy and practice rather than empirical research (see Green, 2016; Madden and Speed, 2017; Beresford, 2019; Paylor and McKevitt, in press). Whilst this points to the dominance of consumerist approaches and a need for more equal relationships, some redistribution of power and a more influential public voice, there is a lack of empirical data to explore the barriers that may prevent this and the tensions and complexities about how this might be achieved. This paper aims to fill this gap by conducting secondary analysis of a commissioned data set generated from semi-structured interviews with researchers and public partners, identified by PI experts as being demonstrably inclusive in their research, to see how this plays out in practice. What is the role of the public partners? To what extent do researchers and public partners perceive that they are operating on an “equal basis” in practice? What facilitates/prevents this happening? How is power expressed and negotiated and is there any evidence of a shift from more hierarchical toward more equal relationships?

## Methods

### Design

We adopted a qualitative inductive approach to achieve a contextualized understanding of the relationship between applied health researchers and public partners. In-depth semi structured interviews with both researchers and public partners enabled us to explore the development, scope and limitations of the relationship established between the two and the negotiation and expression of the power dynamic within the relationship.

### Sample and Participants

The data set was originally collected to inform the development of guidance about co-production being produced by an INVOLVE working group. The authors were commissioned to carry out 10–20 interviews with applied health researchers and their public partners involved in NIHR, or other national peer reviewed funded, research projects in the UK. We specifically sought participants who were identified either by the INVOLVE working group or other NIHR contacts as demonstrating a commitment to inclusivity. This was key to sample selection and was operationalized as public contributors taking part in a range of different activities throughout the life cycle of the project including research design thus requiring the development of an on-going relationship between the professional researcher and public contributor. We also aimed for maximum variability in terms of research topic and research design in order to have representation from those involved in areas such as mental health and qualitative research where the value of experiential knowledge is relatively well-established and those topics and methodologies where it is less so.

As the interviews were conducted, either the participants themselves or others, highlighted other projects which they perceived as demonstrably inclusive or using a different type of research design, and the chief researcher of these projects was subsequently contacted. Thus, a mixture of purposive and snowball sampling was used resulting in a sample of twenty, 14 of whom were professional researchers (including two with lived experience as a service user) and six public partners. We had hoped to include an equal number of researchers and public partners but in most cases the initial contact was with the researcher and getting details from them of public partners who we could contact was not always forthcoming. All interviewees were sent a letter of invitation, participant information sheet and consent form and offered the choice of being interviewed in person, by phone or video call. Verbal consent was recorded at the start of the interview and the project was approved by the University of Essex ethics committee.

The participants are listed in Table 1. The six public partners had all worked with one of the academic research participants (see Table 1). To preserve their anonymity we have not included details of participants' research topic or type of design. In total, there were more female participants (10 female and 4 male researchers and 5 female and 1 male public contributor) which reflects the gender composition of the public involved in research. Project areas included: arthritis, dermatology, two stroke, two musculoskeletal, diabetes, three mental health, dementia, renal, social care, community health. Methodologies used in the projects included four RCTs, two mixed methods, qualitative, systematic review, two feasibility trials, knowledge transfer, experience based co-design, two critical evaluations. Of the public participants, four had professional experience, three in education. All had experience of being involved in NIHR-funded research projects, ranging from large scale multi-site complex clinical trials to individual fellowships. All the six dyads (researcher and public partner) interviewed had a durable long-standing working relationship having worked together on more than one project, generally from the beginning or early stages of designing the project proposal to the dissemination of results.

**Table 1:** List of participants.

**ID**	**Gender**	**Role in research**	**Partners**
**ACADEMIC RESEARCH/CLINICAL PARTNERS (R)**			
R1	Female	Principal investigator/senior research fellow	P6
R2	Female	Programme manager/research fellow	
R3	Female	Principal investigator/professor	P3
R4	Female	NIHR fellow	
R5	Female	Research fellow mental health	
R6	Female	Research fellow mental health	
R7	Female	Principal investigator/professor	P1
R8	Female	Principal investigator/qualitative researcher	
R9	Female	Principal investigator/clinical lecturer	P4
R10	Female	Principal investigator/research fellow	P5
R11	Male	Principal investigator/professor	
R12	Male	Clinical trialist	
R13	Male	Research associate	
R14	Male	Clinical professor	P2
**PUBLIC PARTNERS (P)**			
P1	Female	Mental health service user researcher	R7
P2	Female	Public co applicant	R14
P3	Female	Patient advisory group member	R3
P4	Female	Patient advisory group member	R9
P5	Male	Patient advisory group member	R10
P6	Female	Patient advisory group member/lay researcher	R1

### Data Collection

The interviews were conducted February–April 2017 by one of the authors who is employed as a public involvement lead for an NIHR organization. They were conducted either face to face or via phone or skype using an interview framework informed by an INVOLVE co-production working group and reviewed by public representatives. The interviews asked about specific research projects and the role of the public contributor(s), the relationship between the researcher(s) and the public partner(s), how they conceptualized co-production, and the extent to which the research they were involved with was co-produced, and what barriers did they face when using more inclusive methods of involvement.

### Data Analysis

Interviews were recorded, transcribed verbatim and used initially to inform guidance on co-production produced by the INVOLVE working group (Hickey et al., 2018). Our secondary analysis of the data focused on the identification of salient themes related to the relationship between the researchers and public partners and the power dynamic between them. There were no questions in the interviews specifically about power but this was identified as a key theme in the initial analysis conducted to inform the INVOLVE working group. We coded the data using standard inductive thematic analysis (Braun and Clarke, 2006). A theme was defined as “a patterned response or meaning within the data set” (Braun and Clarke, 2006, p. 11). Codes were grouped together and initial theme titles were generated. This was firstly done independently by the two co-authors to identify preliminary first order codes. These were then shared and discussed at an interpretive level. This focused on discussions about commonalities and differences between the codes of both authors to determine key categories. We explored the meaning of each category and linkages between them to further refine/collapse them to identify core themes relating to the relationship and power dynamic between researchers and public partners.

We were reflexive throughout this process in terms of critically examining potential subjectivities in the data. This included thinking about the impact of the public partners being nominated by the researcher, which will have had a direct impact on the sample as those nominated were likely to have had a positive relationship with the researcher. The fact that the interviewer was a professional PI facilitator is likely to have influenced the content of the interview. We were also aware during the analysis that the lack of a public representative to assist with analysis of the data will have impacted upon the interpretation of the data.

## Results

Having identified the main categories related to the role of public partners, the types of relationship between researchers and public partners and the barriers to operating on an equal basis, we refined these to locate stable key themes to emerge from the interviews (see Table 2). These are discussed below and analyzed to explore the exercise and expression of power.

**Table 2:** Key themes.

**Initial category**	**Final theme**
Role of public partners: they are a necessary and valued part of team with a unique perspective	The value and contribution of public partners
PI not valued by parts of the scientific establishment	
Public partners need support, training and management	A co-opted relationship
Public partners are empowered through their involvement	
Public partners are grateful for the opportunity	
Need to build trust/relationships between researchers and the public partners	Equal partners
Need to share power between researchers and the public	
User led research	A user-led relationship
Governance and bureaucracy of research creates logistical challenges	Constraints/barriers linked to public involvement in applied health research
Research is time pressured and public involvement is time consuming	
Accountability	
Hierarchy of applied health research	

### The Value and Contribution of Public Partners

Both the researchers and the public partners described a range of PI activities and roles. These included the public partner being a named co-applicant on a research project and/or being members of a project steering group. This involved participation in strategic discussions such as priority/research agenda setting and developing outcome measures as well as project oversight (e.g., recruitment monitoring). Public participants were also involved in operational research activities such as: developing research tools; co-designing information sheets for research participants; interviewing research participants (alongside the researcher); helping to interpret the results; presenting the results including co-authoring articles. Two of the public members interviewed had become so involved in these types of activities that they defined themselves as “user researchers.” Most of the public partners were involved in projects directly related to their health condition, but some were also part of a generic research user group.

There was consensus about the inherent value of including public partners in research teams and many examples of it being integral to the research project. According to one dyad (R3 and P3), the public partners give the research credibility as they represented “an authentic voice, they will sit with us, they're on our side” (R3). The public partner felt that their voice was important to ground the research in the lived experience of the public to ensure that it would benefit patients, saying, “we do have a voice and if we think that the research is going off at a tangent or in fact there is not going to be any benefit for Joe Public at the end of it, we will say so, and that has to be taken account” (P3). She explained how the public contributors had been “integral in the design of this study” and felt that “it could not have been done I think without us” (P3).

However, all participants cited examples of some researchers being more skeptical about the benefits of PI. According to the researcher R9, “there's obviously a lot of um varying, still I think acceptance of patient involvement in the clinical research field,” a sentiment echoed by the public participant P5 who noted, “you can feel there are perhaps undercurrents of that, that not everyone thinks that PPI is a good idea.” Some of the research participants attributed this to working in a context where PI was not well-aligned to research timetables and changing research in response to public feedback may result in missed deadlines harming people's research career. As one researcher put it (although he personally did not share this view):

R12: so it's not attractive often, to academic researchers to do this [listen to the public voice], to say well actually let me just get side-tracked here and do….it's a mad idea if you're an academic researcher, it's frankly idiotic, why would you?!

Others reported that PI was not always valued by the scientific establishment. One researcher noted that she had recently submitted a paper to a journal but purposely omitted to mention that it was based on a co-produced survey as “it might even count negatively” (R9). The lack of evidence about the impact of PI in general, and more inclusive methods such as co-production in particular was a barrier. According to R11, “Where's the impact, how am I going to sell this to the chief exec, this is the way you should be working, there is very little in the literature.”

### A Co-opted Relationship

In this theme the public member is framed as slotting into a space created for them by the researcher/research team who assign them a designated role. This includes provision of training and other support to equip them to perform this “co-opted” role. It is well illustrated by the quote below:

R4: Um and the thing about patient representatives coming on to a research panel, … there's often a pre-existing structure for how you manage a research process and the patient representatives are added onto that, what already exists, and they're given training so that they learn how to fit in with the pre-existing structure.

The emphasis here is on how public contributors are slotted into a framework rather than being core members from the start. They are “added-on” and training is positioned as showing the public member how to communicate in an appropriate manner to fit in with “that pre-existing structure.” In so doing, it closes down opportunities for discussion about whether the addition of public members to the research team might open up new ways of assembling the “pre-existing structure.”

This type of co-opted training was also noted by the public participants:

P4: there certainly has been a lot of training and I think it has been done very well and it has been very much appreciated …, but I think the challenge all the time um is whether you are trying to turn patients into mini medics, in other words are you actually trying to make the patients conform to what, y'know what the researchers need, or are you adapting the research?…. um on balance, in the past, it has been very much the former.

This quote with its emphasis on “trying to make the patients conform” suggests an inherent power imbalance. The speaker is explicit that the researchers want the “patients” to “conform” to “what the researchers need” rather than “adapting the research” based on input from the public contributors. She went on to say that she occasionally “has to bite her tongue a bit,” i.e., her voice is contained and constrained.

The co-opted relationship is thus characterized by an unequal power dynamic as the researcher is in control and provides “support” so that the public partner understands and is equipped for their role. It emphasizes how public members are guided and inducted into the research environment, rather than how the public member may shape that environment. From the perspective of the public contributor, this may be represented as alienating, such as in the case below where a researcher's use of medical/scientific language was not understood:

P5: We had a very nice presentation on the value of ablating nodes in fibrillation and I honestly saw a load of my colleagues [other public contributors] wilting under the onslaught.

The assimilation of public partners in a co-opted relationship is generally represented as relatively straightforward. The public partners referred to the skills they had acquired from their professional backgrounds (e.g., as a teacher) as assisting this process and being “really helpful” (P3). However, there were examples of assimilation being problematic:

R2: he [public contributor], was quite spiky about um, being involved, [saying] ‘I don't want to be involved if I'm just a checkbox, I don't want to sit through meetings where I don't understand where any of you are going on about', y'know that sort of thing? …., he's not that communicative, so um, and I sometimes feel like he's getting cross but he doesn't say and then he'll go ‘rahhhh y'know I can't keep up' so um it's quite, he's a more difficult, it's more difficult …. I think if they [the public partners] don't have experience of research, then there does need to be quite a significant training programme.

While this quote is from a researcher who is committed to inclusive methods of PI, she nevertheless locates the problem in the public contributor who is “spiky,” “not that communicative” “more difficult.” For her the solution is to preserve the existing power relations through the provision of more training to help him conform to the role. The problematizing by researchers of views or behaviors of public contributors which do not “fit” their designated role illustrates the unequal power relationship.

A further illustration of a power differential in the co-opted relationship was the way that public partners expressed their gratitude for the opportunities that came with the role. Being a co-applicant, co-authoring a paper, traveling to conferences overseas to give a presentation about the research were hugely valued and appreciated. The public partners said many times that they were “lucky,” with one saying, “I felt very privileged to be able to contribute my opinion.” Somewhat ironically, given the power differential evident in the co-opted relationship, the notion of “empowerment” of public contributors was emphasized. Experiences such as being invited to present to an all-party political group at the UK parliament were reported to be empowering.

The co-opted relationship was described as being akin to that between parent-child and supervisor-student. Take for example, the excerpt below about the researcher trying to get the public partners to conform to the “researcher way” of doing data analysis.

R1: but it was really quite difficult, not difficult, it was quite challenging to keep them [the public partners] on the point…Because they would see things that would start conversations going and we always had limited time….they had to learn over the time that we can't keep adding stuff now….

The emphasis on this being “quite difficult” which was then modified to “challenging” was in reference to the “limited time” available but nevertheless is an illustration of how the co-opted relationship serves to preserve the existing power differential by emphasizing the need to “tame/control” the public voice to meet the research agenda.

### Equal Partners

This theme focuses on the equal value of researchers and public contributors and stresses the need to build bridges to connect their world views in order to share power and decision-making. The added value of the public voice is largely absent from this theme, rather it accentuates the knowledge of all members of the research team which is viewed as having equal value. This was clearly articulated by both researchers and public partners as illustrated below:

R4: …it's basic principles for participatory approaches which is um everybody has an equal seat at the table, um, we try to facilitate environments and settings where people recognize that each other have unique knowledge and skills.

Here, the researcher explicitly mentions having an “equal seat” and emphasizes the “unique knowledge” that each brings. The public partners expressed this as having a different perspective based on their experiential knowledge.

P2: But the whole point for patients, if they want to do this kind of thing, is never to feel as though you are inferior, you're just different, with a different perspective, they are the experts about the disease and all of that, you actually suffer from it, so your input is incredibly important in how they design the trials and the outcomes they expect from the treatment.

The “equal partners” theme was most evident in the interview with a researcher (R4) who had a background in community based participatory research. Her approach began by contacting community organizations to ask them what support and outcomes they would value and then contacting commissioners of services in order to “co-design programme specifications rather than doing a bit of consulting and putting together what they think a service ought to look like.” She highlighted the need for partnership, sharing, respect and developing relationships to establish productive communication between researchers and public partners so that both parties come to understand the perspective of the other. Training is still required but unlike the “co-opted relationship” where the public need to be trained in the world view of the researcher, in the “equal partners” theme, everyone needs training to work together and “see through the eyes of somebody in a different culture.”

R4: And what I'm trying to do is build little bridges across them … so that they know how to communicate and get along or y'know bringing academics into a community setting and training them so they know how to get along….so that they start to look at the issues through the lens of the other person… to be able to see something through the eyes of somebody that's in a really different, different organizational culture.

Participants explicitly acknowledged the need to challenge and change existing power relations and hierarchies to achieve a shared space:

R6: I think a lot of it is about respect and sharing, sharing the space so sharing the voice in terms of design and respect for other people's opinions, um, I think um it's a partnership…. so I think it's about breaking down a hierarchy so if you are going to do any research that's co-produced, it can't have a hierarchy there, um, it has to be equal value.

Being “equal partners” was seen as important for co-production, which was framed not only as different from traditional or mainstream patient and public involvement (PPI), but also as radical and creative:

(R11) Well, I think um, I think PPI has become sort of mainstream and for me, co-production is, if it's not radical then there is nothing to it, it's that radical element of bringing service users, providers together um to shape the delivery of services and ……I mean one of the things about co-production, co-design is you never know, quite know where these projects are going to end up, that's the whole point, they are creative, they are emergent.

Thus, the equal partners theme highlights a fresh and radical approach in which researchers and the public share power.

### User-Led Relationship

There were two examples of public partners initiating and leading or co-leading projects. In both cases they were motivated by their own experiences as service users and were not representing a user organization. The user-led relationship theme can be viewed as an extension of the equal partners theme and there is evidence of a discernible shift in the power dynamic between the public partner and the researchers. In the first case the public partner approached a researcher about the lack of evidence about her condition and following this became the co-ordinator for a systematic review. She clearly affirmed “I am the lead …No question, they came to me, I dished out the work, I told them the deadlines, I organized the whole thing, I didn't do the work” (P2). She drew upon her organizational and librarianship skills developed in her working career as well as her lived experience of the condition to drive the project and this was confirmed by her research partner (R14). She described her relationship with the researchers as “Equal, equal footing, great respect on both sides, no condescension.” Her selection of the phrase “no condescension” suggests that there is an expectation that professional researchers may sometimes adopt a patronizing attitude toward public partners. She was clear about when her input was and was not required saying, “There are times when I can't have any input, when it comes to statistics y'know, forget it, I haven't got a clue, or the methodology, but there are times when what I think will make a difference or will make them rethink.”

In the other case (P1), the public partner had the initial idea for the project and contacted the director of services, who responded enthusiastically, and they became the co-leads. The public partner reported that it took time to establish a relationship and break down the power differential and that this process was initially daunting:

P1: Um, so in that first meeting [with the Director of acute services], I think it's fair to say I felt pretty um nervous, um because obviously the power differential was huge y'know um, he was someone in considerable power and I was someone who didn't have that sort of feeling. Um, so I went into that room feeling very, I remember feeling very anxious um and so it took a while before I really felt um that we established a relationship where we were more, much more on an equal footing.

This illustrates how power relations are negotiated and performed between someone perceived as having “considerable power” in comparison to her own as she “didn't have that sort of feeling.” She had to overcome anxiety and persevere over time to establish being “much more on an equal footing.” The qualifier “more” rather than being “on an equal footing” suggests that some power differential may still exist.

### Constraints Linked to Applied Health Research

This theme relates to applied health research and in the interviews was interwoven with both the co-opted and equal partners themes. It was present in interviews with all the participants but most prominent in interviews with researchers. It was used to frame narratives to explain the challenges, constraints and limits of PI in research. These constraints included: barriers linked to the governance and bureaucracy of research; the imperative of research protocols and deadlines; the hierarchy of applied health research; and issues around accountability for the research.

Research governance procedures were reported to be bureaucratic and time consuming requiring negotiation with a complex field of actors and processes:

R6: we have found one of the major challenges was, with involving service users in the actual data collection is all of the um pre-engagement checks and DBS [Disclosure and Barring Service to check for criminal record] and all of those kind of things, they're a massive hurdle, trying to get research passports is, does take a prolonged amount of time, maybe 6 to 9 months in some cases ….it does become a case of banging your head against the wall….

In this case, the governance requirements resulted in some of the public partners being unable to take part in the project.

Another feature of research, which acted as a constraint, was the imperative of following a detailed and complex research protocol and working to tight deadlines. One participant talked about the, “complex day-to-day running of things which you can't practically involve with at every step…I am thinking of big trials I'm involved in….Y'know the machine that is the clinical trials unit” (R9). The time required to build relationships with public partners and involve them in all decisions is not compatible with the operation of the clinical trial “machine.” All the researchers were adamant that “at the end I have to produce a report, I have to make my deadlines to my funder” (R10).

The hierarchy that privileges scientific knowledge associated with much applied health research was also constraining. This is well-illustrated by a researcher (R5) who said she was committed to: “power sharing” and “doing things differently” but was thwarted “cos research doesn't seem to think, doesn't look like that…It's a very hierarchical environment to work in so when you then introduce um co-production into it, it's really difficult I think.” She related the following experience:

R5: the research team had basically decided that …they [the service user advisory group] had done really good work but now they really didn't want them to meet very often and the advisory group were really, really passionate about this topic and …they wanted a bigger role … I felt stuck between the team who I knew were really busy um and just want to get on, collect data and a passionate advisory group that really can't be tokenistic otherwise just don't bother with us and I was stuck in the middle.

This illustrates the power differential and constraints of PI in health research. Having been mobilized the user group want to carry on influencing the research whereas the researchers feel that this would not add value. The user researcher who coordinated the user group was indeed “stuck in the middle.”

A further constraint was accountability. This was rarely raised in relation to public contributors and when it was there was a generalized acceptance that they could and should not be accountable for the research. This was even the case when a public partner was named as a co-applicant suggesting that this status was mainly tokenistic. The quote below from a public participant explicitly links accountability to the power dynamic:

P4: very conscious of the unequal power relations and um, but in a way, that's, at, in some ways, that's as it should be because of the accountability factor and um, it is at the end of the day, it's the researchers and the medics and er, y'know they are the people that are accountable for the research that they do and um y'know we are, y'know we make a voluntary contribution and I think it is very, very important that that is increased and is listened to but we can't be held accountable for the outcome of the research and therefore that, that does make co-production a slightly problematic concept.

Accountability here is identified as a key issue in power-sharing as it involves a sharing of responsibility and in the quote above it uses this to problematize co-production and power-sharing.

## Discussion and Conclusion

The findings of our research clearly indicate that, in the area of applied health research, both professional researchers and public representatives place a high value on the integration of scientific and lay perspectives. There was also evidence that it is clearly possible for public partners to feel sufficiently empowered to voice their opinions and play a significant role in decision-making in areas where expertise based on experience is demonstrably useful. In these contexts, our participants reported working together synergistically in co-opted, equal partner and user-led relationships. In their accounts they talked about producing high quality research using and blending the unique types of knowledge, experience and perspective held by all team members. However, some clear power differentials were apparent in their accounts and the narratives of both researchers and public partners suggested that their collaboration was characterized by the “co-option” of patient and public representatives into a professional/scientific framework, rather than vice-versa. It should also be noted that some of the participants in our project felt that the wider research establishment is not yet ready or able to accept that “experiential knowledge” is a distinct way-of-knowing which merits parity or equality with “scientific knowledge.”

Our findings suggest that this continuing “inequality” is not necessarily due to the researchers being unwilling to share power. It should be remembered that our research participants were purposively selected on the grounds that they were identified by colleagues as being involved in projects known to be enthusiastic about public participation. It follows then that the people we gathered data from tended to be committed to a progressive involvement agenda. Specifically, they were committed to:-strengthening public voices; sharing power over decision-making and; trying to achieve “co-production” in research (although not all of them used this terminology). That even this identifiably “radical” group struggled to demonstrate an equal power dynamic throughout the research process suggests that significant challenges and barriers are inherent in the development of these ways of working.

One key barrier is the “positivist paradigm” that predominates in applied health research. This overall philosophy tends to privilege classically scientific ways of thinking such as structured sampling and standardized measurement, thus creating significant difficulties in articulating and including lay views (see Taylor, 2007). This happens because “experiential knowledge” is by its very nature based on individual perception and observation and thus easily characterized as at best “***sui generis***” and at worst “anecdotal.” Concretely in our data this tendency to perceive experiential knowledge as problematic from a methodological point of view is discernible in the fact that all the researchers we spoke to emphasized the importance of a “hierarchy of research” when explaining why public involvement in general, and the establishment of equal relationships in particular, is challenging. In this light, our analysis highlights how the sharing of power is generally incompatible with the existing research hierarchy, which is why co-opted relationships are most common.

In addition, the time taken to build and maintain relationships is often compromised by the imperative to follow research protocol deadlines. Neither is there a clear mechanism for public partners to take accountability for research as they generally do not routinely have access to protection such as insurance indemnity which is available to academic researchers from their employing institutions. The principles of inclusivity and “sharing power and responsibility” which are central to the definition of co-production (Hickey, 2018; Hickey et al., 2018) therefore do not fit with the dominant research culture which remains a hierarchical environment in which researchers are at the top and public contributors are at the bottom (Crowe and Giles, 2016). This tends to frustrate the process of the “conscientization” of the public through participation mentioned in the introduction to this paper.

Our analysis thus provides empirical evidence supporting the theoretical argument put forward by others that the way that research is organized creates a barrier to more inclusive methods of involvement. This in turn suggests that a major shake up of research practices, culture and hierarchies may be required for power sharing to become a reality (Hickey, 2018; Wicks et al., 2018). The notion of co-production and inclusivity arises from a tradition that sees people as assets of equal worth. It thus has an uneasy fit with the hierarchical model of scientific research. The culture of research therefore needs to change and embrace more democratic participatory approaches. There are examples of such approaches from the health user movement (Beresford, 2019), such as emancipatory disability research (Barnes C., 2003) and “Mad studies” (Beresford, 2016) where users have challenged the hierarchy of the research community which privileges scientific knowledge. This does not mean that methodologies and research plans based on a traditional positivist approach should be abandoned, but rather that to attain equal relationships in research we will need to broaden the range of research approaches supported by funding agencies such as NIHR. A greater focus on community and social care research will likely require different methodologies and more participatory approaches and it is within these ways of working that public partners can more readily share power. However, whether such a culture shift is achievable given the wider neoliberal forces and consumerism that dominates the implementation of PI in applied health research is uncertain.

## Data Availability

The datasets for this study will not be made publicly available because Interviewees were not informed that they would be publicly available.

## Author Contributions

TJ initiated the study and its design, conducted the interviews, collated all the data, and identified the preliminary themes. GG and TJ discussed the interpretation of the analysis, contributed to this study, read and approved the content. GG drafted the paper.

### Conflict of Interest Statement

The authors declare that the research was conducted in the absence of any commercial or financial relationships that could be construed as a potential conflict of interest.
